# Efforts to Enhance Recruitment and Engagement of Caregivers from Medically Underserved Communities in a Randomized Controlled Trial of a Vaccine Promotion App

**DOI:** 10.1017/cts.2025.10164

**Published:** 2025-11-06

**Authors:** Erin Dawley, Jonathan Figliomeni, Russell McCulloh, Ellen Kerns, Songthip Ounpraseuth, Di Chang, Kristina Foster, Christine Hockett, Karlyn Martini, Melinda Delaney, Angel Munoz Osorio, Michael Nelson, Katie Queen, Daniel Blatt, James R. Roberts

**Affiliations:** 1 Medical University of South Carolina, Charleston, SC, USA; 2 University of Nebraska Medical Center, Omaha, NE, USA; 3 University of Arkansas for Medical Sciences, College of Medicine, Little Rock, AR, USA; 4 University of Kansas Medical Center, Kansas City, KS, USA; 5 University of South Dakota School of Medicine, Avera Research Institute, Sioux Falls, SD, USA; 6 Dartmouth Hitchcock Medical Center, Lebanon, NH, USA; 7 The Warren Alpert Medical School of Brown University, Providence, RI, USA; 8 University of Cincinnati, Cincinnati, OH, USA; 9 University of Kansas School of Medicine, Kansas City, KS, USA; 10 Our Lady of the Lake Children’s Hospital, Pennington Biomedical Research Center, Baton Rouge, LA, USA; 11 University of Louisville School of Medicine, Louisville, KY, USA

**Keywords:** Representativeness, COVID-19, clinical trial, mobile health, health promotion

## Abstract

Over 15 million children in the United States have been infected with COVID-19; nearly 2,000 have died. Approval of COVID-19 vaccines for children enabled reductions in disease severity and mortality. Disparities in vaccine adoption exist along racial, ethnic, and rural–urban lines, with lower uptake among medically underserved populations (e.g. Black, non-Hispanic White rural populations) compared to urban White populations. This study examined efforts to recruit and engage a diverse cohort as part of a vaccine communication randomized trial conducted across 15 states and compared demographic characteristics of the enrolled cohort to the broader US population. To enhance recruitment of diverse populations, eligible clinics had to serve a significant proportion of medically underserved individuals based on race, ethnicity, or geographic location. Coordinators used both traditional (in-person daily clinic schedule review) and retrospective (EHR and billing data review) recruitment methods adapted to enrich engagement with focus populations. Demographic characteristics were compared to national statistics obtained from the CDC’s Household Pulse Survey. In total, 2999 parents/caregivers were screened; 725 were randomized (24.1%). Comparing enrolled subjects to the demographics of participating states, 17.3% vs 9.8% self-identified as Hispanic, 39.6% vs 13.0% as Black. Additionally, 34.3% self-described as living in a rural area. Of the 725 randomized, 512 (70.6%) completed the baseline survey. Of these 512, 422 (82.4%) also completed the final survey of the 24-week study. This analysis demonstrates the Institutional Development Award States Pediatric Clinical Trials Network can successfully recruit and engage populations from diverse and underrepresented populations in research.

## Introduction

Since the SARS COVID-19 (severe acute respiratory syndrome novel coronavirus) pandemic began in early 2020, more than 15 million children in the United States have been infected, and nearly 2,000 children have died from a SARS-CoV-2 infection [1,2]. For comparison, there have only been 436 reported deaths due to influenza virus among children since 2020 [3]. From December 2020 to May 2021, the US Food and Drug Administration (FDA) expanded the Emergency Use Authorization (EUA) of the Pfizer and Moderna COVID-19 vaccines from adults to adolescents 12 years of age and older. The EUA for these vaccines was expanded further in December 2022 to include children and adolescents as young as 6 months of age [4]. The messenger ribonucleic acid (mRNA) COVID-19 vaccines are 50%–90% effective at reducing the severity of symptomatic infection and demonstrate almost 100% protection against severe disease and death [5,6]. Despite the effectiveness of COVID-19 vaccines against severe infection and death, individuals from medically underserved communities – including Black and rural communities, and socioeconomically disadvantaged populations resulting from systemic and institutional marginalization – have disproportionately lower COVID-19 vaccination rates [7,8], which contributes to the disproportionately higher disease and mortality burden experienced among individuals from these communities as compared to those from urban, White non-Hispanic communities [9–11].

The Health Belief Model provides an organizing framework for examining factors contributing to lower vaccine uptake [12,13]. In particular, sociodemographic factors are associated with COVID-19 vaccine uptake, including age, rurality, and race/ethnicity [14]. Since the EUA was issued for children as young as 6 months, only 25% of children ages 6 months to 4 years of age have received 1 dose of the COVID-19 vaccine in comparison to 47% of 5- to 11-year-olds and 77% of 12- to 17-year-olds [14]. COVID-19 vaccine uptake has dramatically lagged in rural communities when compared to urban communities. On August 11, 2021, a nearly 14 percentage point gap had emerged between adults in rural counties (46% fully vaccinated) compared to 60% in urban counties [15]. By January 2022, the gap remained, even among those who had received only a single dose: 59% of the rural population versus 75% of the urban population [16]. Disparities in vaccine uptake also exist by race and ethnicity. For example, Black communities have demonstrated lower confidence in COVID-19 vaccines due to institutional mistrust, and uptake among Black individuals still lags compared to those who identify as non-Hispanic White. [17–19] While a long – and occasionally horrific – history about medical research in the Western world is chronicled by expert medical historians, an abridged version prior to the advent of Institutional Review Boards can be summarized thusly: ethical lapses by medical practitioners [20], public health professionals, and the civil servants engaging in atrocities ranging from surgery without anesthesia [21] to the systemic infliction of preventable suffering on indigenous and other marginalized peoples [22] have justifiably led surviving communities to be skeptical and distrustful of research and the medical community.

**Figure 1.**
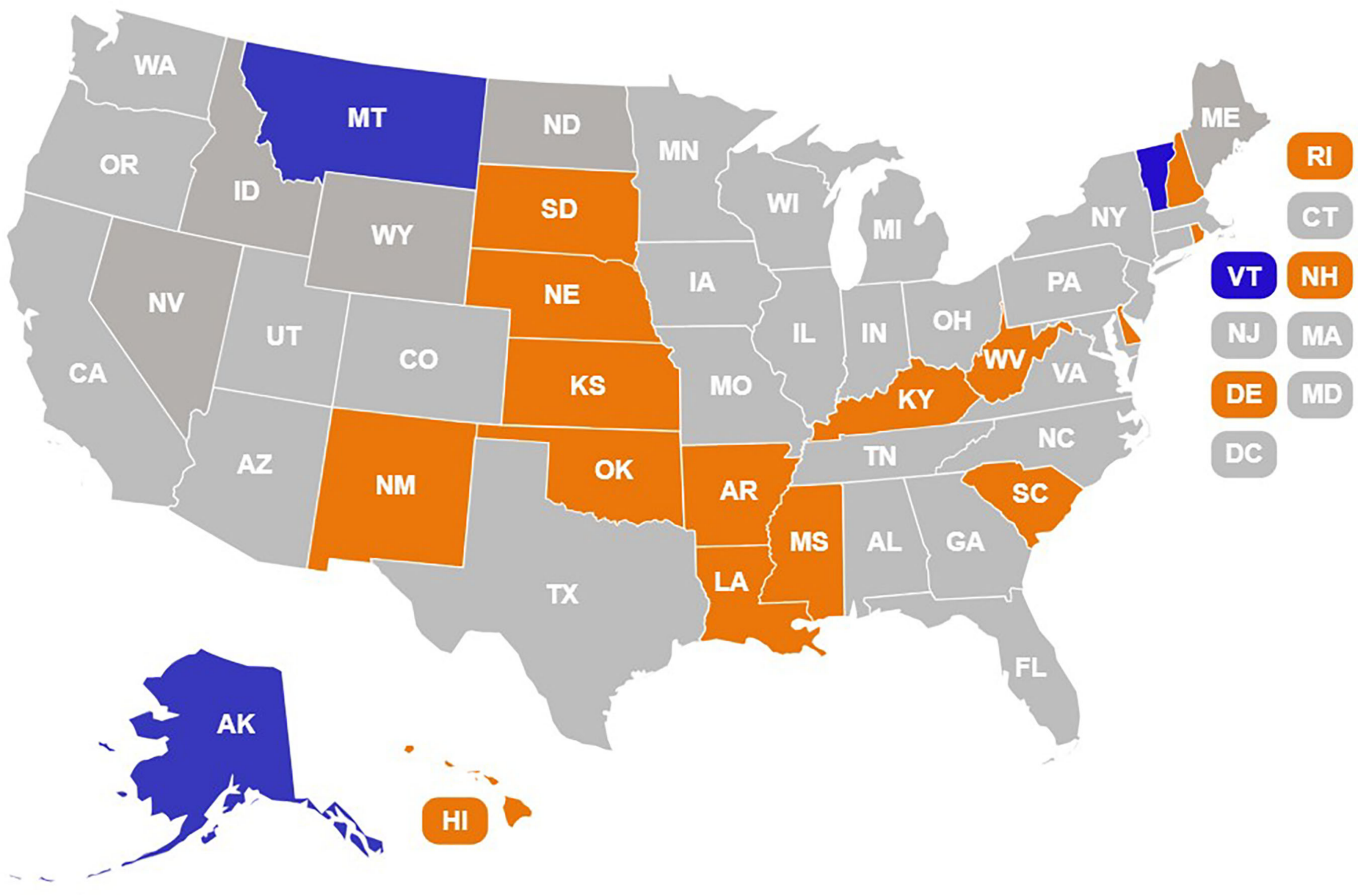
Geographic reach of MoVeUP^Þ^ Study and ECHO^ð^ IDeA States Pediatric Clinical Trials Network. ^Þ^Improving pediatric COVID-19 vaccine uptake using an mHealth (mobile health) tool (MoVeUP). ^ð^Environmental influences on Child Health Outcomes (ECHO).

According to the National Institutes of Health (NIH), there is a need to tailor vaccine interventions to specific target audiences to effectively increase vaccine uptake and overall confidence in vaccines among these populations. The NIH-funded Environmental influences on Child Health Outcomes (ECHO) Institutional Development Award (IDeA) States Pediatric Clinical Trials Network (ECHO ISPCTN) is comprised of 18 sites from geographically diverse areas of the United States (Figure 1) [23]. The Improving Pediatric COVID-19 Vaccine Uptake using an mHealth (mobile Health) tool (MoVeUP) clinical trial was a multi-site, randomized controlled trial conducted by ECHO ISPCTN that aimed to determine the effectiveness of a customized vaccine communication app developed using Agile Software Design approaches with intervention elements organized under the Health Belief Model and delivered to parents/caregivers of children unvaccinated against COVID-19 [24]. The intervention app was customized based on rurality and race/ethnicity, with tailored content based on the results of the qualitative sister study previously conducted by the network (MoVeUP Qualitative) [25]. The previous qualitative study interviewed individuals who identified as being from one or more race, ethnic, or geographic background associated with low COVID-19 vaccine uptake. These underserved populations (rural, Black, Hispanic, and non-English speaking) are commonly underrepresented in clinical research due to procedural and psycho-social barriers that come with recruiting for clinical trials [26,27]. Results from MoVeUP Qualitative identified specific concerns related to COVID-19 vaccination in children [25]. Researchers across the ECHO ISPCTN and others have identified commonly reported challenges to clinical trials participation encountered in these underrepresented research populations, including a lack of access to health care, distrust in scientific institutions, and misperceptions about clinical research [27–30]. Thus, there exists a gap in knowledge as to how to effectively recruit and engage historically underserved populations in public health research, including vaccine research [31,32].

The purpose of this paper is to: (1) describe the efforts employed to enhance recruitment of diverse and underrepresented populations into the MoVeUP clinical trial; (2) describe the sociodemographic characteristics of enrolled participants; (3) compare these characteristics with population estimates of the states participating in the ECHO ISPCTN and nationally; and (4) describe the engagement of participants beyond simple retention for the duration of the study. To that end, we seek to answer two questions: (1) In what ways were we successful (or not successful) in enrolling and engaging participants from sociodemographic backgrounds associated with lower COVID-19 vaccine uptake?; and (2) How representative was our cohort to that of the broader population?

## Materials and methods

### Clinic selection and participant eligibility

Full protocol details are described elsewhere [24]. Briefly, MoVeUP enrolled participants from primary care clinics within the ECHO ISPCTN (Figure 1). To participate, clinics had to provide primary care to at least 100 unique pediatric patients per year, use an Electronic Health Record (EHR), be able to access their state vaccination registry, and meet at least one of the following diversity, equity, and inclusion metrics for the pediatric patient population they served: (1) <60% non-Hispanic White; (2) >40% from a rural ZIP Code as defined by Rural-Urban Community Area (RUCA) code ≥ 4; and/or (3) >40% with Medicaid or uninsured [33].

To participate, eligible parents/caregivers were those who: (1) were age of majority, as defined by their state of residency; (2) had primary medical decision-making and legal authority to consent to vaccinate for at least one child eligible to receive COVID-19 vaccine but was unvaccinated against COVID-19 at the time of consent; (3) had access to a mobile device that could store and run the study app for at least 24 weeks; (4) were able to speak and read English or Spanish; and (5) their eligible child received primary care services at one of the clinics participating in the study. Internet access was not an inclusion requirement and was not necessary for app usage after the initial app download. Devices that could run the app included smart phones and tablets that ran either the Android or iOS operating systems; smart phone adoption among US adults was 85% in early 2021 [34]. Parents/caregivers and children were not eligible to participate if the child(ren) had contraindications to the COVID-19 vaccine, had participated in a past or current COVID-19 study, or if the child was scheduled or planned to receive the COVID-19 vaccine at the time of enrollment (all assessed by parent/caregiver/self-report). Parents/caregivers were also excluded from participation if they had a cognitive impairment that would limit their ability to engage with the app content and/or make medical decisions regarding vaccination. This criterion was based on the site investigator’s assessment and local human subjects research policies.

### Recruitment methods and participant engagement

Study coordinators could use two different recruitment methods. One approach was the retrospective recruitment method, whereby coordinators identified and contacted potential participants by reviewing a list of patients derived from billing records or EHR visit reports from the previous 12 months. This method has proven successful in recruiting rural parents/caregivers and children in a previous behavioral intervention clinical trial focused on pediatric obesity that was conducted through the ECHO ISPCTN [35]. Study coordinators preferentially contacted families with children with more recent visits as those were considered most likely to be continuing their primary care at the participating clinic. To prioritize the recruitment of participants from communities facing health disparities, the contact list order was adjusted so that patients from rural and/or reported race/ethnicity other than non-Hispanic White were contacted first. Prior to making retrospective recruitment calls, opt-out letters were sent to each patient on the list, via mail or electronically, to give them the option to decline by contacting study staff.

The second method of recruitment was clinic-based, in-person recruitment (referred to as the “traditional method”) [35]. The traditional method has also been proven to be a successful recruitment tool for research participants within the study’s population of interest [36,37]. Briefly, study coordinators previewed daily clinic schedules and identified children who would meet study eligibility criteria. Study coordinators then conducted in-person recruitment and completed informed consent either in-person or via telephone follow-up with interested caregivers. Healthcare providers could also refer caregivers to the study at appointment visits. Caregivers could also self-refer after exposure to study announcements and advertisements shared via EHR messaging, printed flyers, emails, and social media distributed in collaboration with the local clinic. All recruitment materials were provided in both English and Spanish, and all eligible caregivers, regardless of race/ethnicity and rurality, were approached by study teams when conducting in-person recruitment activities.

For both retrospective and traditional recruitment, coordinators would introduce the study and assess interest in participation. If the caregiver was interested in participating in the study, the coordinator would then screen the caregiver for eligibility. If eligible, the coordinator would then complete the Enrollment Survey and randomize the participant using the Research Electronic Data Capture software (REDCap version 12.0.12) [38] to either the general health app (control), which included general pediatric health information, or the intervention app, which included information on COVID-19 vaccines, vaccination education, locations to be vaccinated, a personal message from the pediatric provider in the clinic, etc. Further details regarding intervention and control apps are detailed elsewhere [24].

Irrespective of recruitment method, randomized participants were regularly engaged by their site’s study coordinators. Starting with assistance to download the app itself and completing a first session, to completion of the baseline and subsequent surveys, study coordinators endeavored to maintain meaningful engagement between participants and the study. Throughout the duration of the study, coordinators would make three attempts to contact participants (via telephone, text message/SMS, and/or e-mail) to encourage survey completion; to further promote engagement, participants were remunerated $20 for each completed milestone survey.

### Recruitment monitoring, feedback, and peer-mentoring

Study teams met via teleconference at least weekly to review overall enrollment progress during the active enrollment period. Enrollment progress was also summarized, and reports were distributed to all study team members via email at least weekly. Multiple sessions hosted by protocol leadership and the Data Coordinating and Operations Center (DCOC) focused on sharing successes and challenges to study recruitment and disseminated effective strategies from high-performing sites. Site managers also met with study Principal Investigators and coordinators at least monthly to identify and address challenges to study enrollment and to discuss efforts to engage diverse populations during recruitment.

### Community engagement and development of recruitment materials

The DCOC maintains contact with several community advisory boards (CABs) that provide consultation regarding study design, recruitment, retention (the act of remaining in a study with or without interaction with study activities for its duration), and engagement (the consistent, meaningful interaction with a study throughout the milestones/duration of said study) practices. The CABs were also consulted about best practices for communication with potential participants, enrolled participants, and the broader community. Additionally, the network’s Engagement Working Group, comprised of researchers with experience in health equity and engaging rural and underserved populations, reviewed and provided guidance for the development of study protocols, recruitment materials, and the generation of lay summaries and public-facing education. The goal of this group is to ensure that materials are easily understood and acknowledge cultural and sociodemographic contexts relevant to trials being conducted by the network. One of the protocol chairs (RM) also served as a member of the Engagement Working Group and organizes a CAB comprised of healthcare providers, public health workers, parents/caregivers, community activists, and individuals from rural and Hispanic communities in Nebraska (one of the states participating in the ECHO ISPCTN). This Nebraska CAB was consulted during the development of the MoVeUP protocol and provided formative feedback on study design and participant recruitment. Recruitment materials and consent documents underwent DCOC CAB and Working Group review and edits to maximize acceptability to individuals from priority populations.

### Data sources

Demographics of enrolled caregiver participants and the eligible unvaccinated children were collected after informed consent. We used the US Census Phase 3.7 Household Pulse Survey (PS) to obtain sociodemographic information on the population of children and adults unvaccinated against COVID-19 in the United States overall and specifically within the IDeA States. This survey is an experimental data product and is an Interagency Federal Rapid Response Survey to Measure Household Experiences during the Coronavirus (COVID-19) Pandemic. The PS is conducted by the United States Census Bureau in partnership with 15 other Federal agencies and designed for quick and efficient deployment of survey tools for collecting data on emergent issues impacting US households [39]. While the focus of these surveys has historically been social and economic in nature, the Centers for Disease Control and Prevention (CDC) through the National Center for Health Statistics has included health-related questions in the Household Pulse Survey since April of 2020. The analyses for the present study used the week 53 survey in which data were collected from January 4–January 23, 2023. The replicate weights provided with the survey were used in the statistical analyses.

### Analysis

We used the MoVeUP Parent/Caregiver sociodemographic survey results to describe absolute counts and proportions of racial, ethnic, and rural participants (Table 1). The Household PS data is summarized and described for all IDeA States and non-IDeA States with percentages accounting for replicate survey weights. Additionally, we used unweighted chi-square tests to examine the distribution of key demographic characteristics between parent/caregiver participants in the MoVeUP cohort compared to all IDeA States and non-IDeA States based on the Household PS data.

**Table 1.** MoVeUP^Þ^ parent/caregiver demographics and national survey based on Pulse (week 53)

Demographic information	MoVeUp^Þ^ randomized (*n* = 725)	National Survey ISPCTN^ß^ States*	National sSurvey Non-ISPCTN^ß^ States*	Unweighted chi-square test *p*-value, ISPCTN States^c^	Unweighted chi-square test *p*-value, non-ISPCTN States^d^
Gender, N (%)^†^				<0.0001	<0.0001
Male	43 (5.9)	(41.3)	(41.1)		
Female	681 (94.1)	(58.7)	(58.9)		
Ethnicity, N (%)^†^				<0.0001	0.0013
Hispanic	125 (17.3)	(9.80)	(21.9)		
Non-Hispanic	598 (82.7)	(90.2)	(78.1)		
Race, N (%)^†^				<0.0001	<0.0001
White	346 (52.3)	(75.0)	(72.9)		
Black or African American	262 (39.6)	(13.0)	(16.6)		
Asian	4 (0.6)	(2.3)	(3.4)		
Other	50 (7.6)	(9.6)	(7.1)		
Caregiver education, N (%)^a^^†^				<0.0001	<0.0001
≤ High school or GED completed	154 (31.3)	(44.5)	(43.1)		
Some college/technical/vocational	202 (41.1)	(35.2)	(32.7)		
Bachelor’s degree or higher	136 (27.6)	(20.2)	(24.1)		
Rurality (self-report), N (%)^‡^					
Yes	249 (34.3)				
No	476 (65.7)				

^Þ^Improving Pediatric COVID-19 Vaccine Uptake using an mHealth (mobile Health) tool (MoVeUP).^**ß**^Institutional Development Award (IDeA) States Pediatric Clinical Trials Network (ISPCTN).

* Percentages accounting for survey weights.

a Only available among participants who completed a baseline survey (N = 512).

^†^Contain missing observations (gender has 1 missing observation; ethnicity had 2 missing observations; race had 63 missing observations; caregiver education had 20 missing observations).^‡^The Pulse Survey did not include self-report rurality.

c Unweighted chi-square test *p*-value (MoVeUP vs National Survey ISPCTN States).

d Unweighted chi-square test *p*-value (MoVeUP vs National Survey Non-ISPCTN States).

## Results

MoVeUP enrolled participants from primary care clinics across 15 sites participating within the ECHO ISPCTN located in the following states: Arkansas, Delaware, Hawai’i, Kansas, Kentucky, Louisiana, Mississippi, Nebraska, New Hampshire, New Mexico, Oklahoma, Rhode Island, South Carolina, South Dakota, and West Virginia. In total, 29 primary pediatric care clinics participated as enrollment locations.

In total, 2,999 parents/caregivers were screened from July 2022 to February 2023. Of those screened, 2,270 failed screening for reasons that included “declined,” “ineligibility,” or “eligible, but declined to consent” and most often citing “not interested,” “no time” or “do not like the COVID-19 vaccine” as reasons; 2 passed screening but withdrew before randomization; and 2 were randomized in error and subsequently removed from the study and related analyses. In total, 725 eligible participants were randomized (24.1%). Among the 725 randomized parents/caregivers, 17.3% (*n* = 125) self-identified as Hispanic (versus 9.8% of National Pulse Survey – ISPCTN states), and 39.6% (*n* = 262) identified as Black (versus 13.0% National Pulse Survey – ISPCTN states). In addition, more than 1 in 3 parents/caregivers self-identified as living in a rural area (34.3%, *n* = 249) as compared to the national 20% of the population that lives in a rural area (Table 1) [40].

Table 2 shows the MoVeUP participants’ engagement for the survey milestones. A total of 70.6% (*n* = 512) of those randomized completed the baseline survey. Of the 512 participants who completed the baseline survey, 82.4% (*n* = 422) completed the final study survey prior to the conclusion of the 24-week study period. Additionally, *rural* participation among both the randomized participants generally and those that completed the baseline survey specifically remained well above the percent of the national population living rurally: 32.4%–35.5% across all randomized, and 27.8%–35.0% across all participants who completed the baseline survey.

**Table 2.** MoVeUP^Þ^ participant milestone engagement of selected race/Ethnicities

	Completed among those that completed baseline *N* = 512		
	*N*	Overall % at each assessment period	Weighted % out of total *N* = 512
**Baseline**	**512**	**100.0**	**100.0**
*Race/ethnicity*			
Hispanic (any)	74	14.5	14.5
Non-Hispanic, White	238	46.5	46.5
Non-Hispanic, Black	171	33.4	33.4
* Rurality*			
Rural	179	35.0	35.0
Urban	333	65.0	65.0
**Week 8**	**469**	**91.6**	**91.6**
* Race/ethnicity*			
Hispanic (any)	64	13.7	12.5
Non-Hispanic, White	221	47.1	43.2
Non-Hispanic, Black	156	33.3	30.5
* Rurality*			
Rural	168	35.8	32.8
Urban	301	64.2	58.8
**Week 16**	**432**	**84.4**	**84.4**
* Race/ethnicity*			
Hispanic (any)	59	13.7	11.5
Non-Hispanic, White	203	47.0	39.6
Non-Hispanic, Black	146	33.8	28.5
* Rurality*			
Rural	148	27.8	28.9
Urban	284	72.2	55.5
**Week 24**	**422**	**82.4**	**82.4**
* Race/ethnicity*			
Hispanic (any)	62	14.7	12.1
Non-Hispanic, White	197	46.7	38.5
Non-Hispanic, Black	142	33.7	27.7
* Rurality*			
Rural	146	34.6	28.5
Urban	276	65.4	53.9

^Þ^ Improving Pediatric COVID-19 Vaccine Uptake using an mHealth (mobile Health) tool (MoVeUP).

## Discussion

The MoVeUP clinical trial successfully achieved the goal of recruiting and engaging caregivers from low-vaccinating populations across geographically and culturally diverse primary care settings. This trial also successfully engaged and retained those participants throughout the duration of the study. The study population included a higher proportion of caregivers who identified as being from Black and rural communities than the national demographic proportions and a higher proportion of Hispanic caregivers than the population average across IDeA states. These findings demonstrate that the MoVeUP study design enabled recruitment diversity along both a racial-ethnic axis (more than 40% of individuals self-identifying as a race/ethnicity other than non-Hispanic White) and an urban–rural axis (more than 30% of participants self-identifying as living in a rural community), and the results highlight the success of the recruitment and engagement strategies employed during the trial to enroll a diverse participant cohort.

Our results provide critical insight into the effectiveness of recruitment strategies to optimize equity of engagement in clinical trials. First, MoVeUP used recruitment approaches first evaluated in the prior ECHO ISPCTN iAmHealthy clinical trial [35]. As in iAmHealthy, MoVeUP study’s active recruitment strategy prioritized eligible caregivers from populations commonly underrepresented in clinical research higher on the contact list, thus ensuring contact with more caregivers from rural or underserved communities early in the enrollment period. Results from MoVeUP lend further support to the effectiveness of this recruitment strategy to ensure diverse enrollment. Moreover, our results demonstrate how equitable access to clinical trials can be achieved by setting clinic inclusion criteria that encourage engaging primary care practices that serve significant proportions of individuals from rural and other medically underserved communities.

MoVeUP’s approach to diverse participant recruitment reflects best practices for establishing recruitment goals. Specifically, the trial’s bundled approaches of community engagement, consultation, clinic selection, iterative feedback from clinical trials teams across the enrollment period, and weekly check-ins with sites to review, interpret, and act upon recruitment and retention/engagement progress reflect recently published recommendations aimed to ensure diverse recruitment [41]. Another recently published framework developed by Pfizer builds upon this guidance by recommending that representativeness be intentional and reflect the populations affected by the condition of interest for the trial [42]. Such engagement aligns well with MoVeUP’s use of the Health Belief Model which highlights the importance of considering demographic and social factors when developing and testing public health interventions intended to reach underrepresented or otherwise medically underserved populations. Further, the success of MoVeUP to both *recruit* and maintain *meaningful engagement* with populations historically underrepresented in research on a topic of such social and medical gravitas illustrates that efforts prioritizing recruitment of relevant populations can be effective *even when* those relevant populations are also locally underrepresented.

The MoVeUP trial and recruitment approaches also reflect successful attempts to overcome barriers to clinical trial participation identified through prior research conducted by ECHO ISPCTN [23–26,35]. Recruitment materials were developed and revised with feedback from multiple CABs comprising individuals from focus sociodemographic backgrounds, thus maximizing the acceptability of messaging to potential participants. Materials were also immediately available in English and Spanish, unlike many clinical trials where translated materials are often delayed or unavailable.

Several limitations in this study offer opportunities for future research in this area. First, study coordinators’ tasks related to participant onboarding and engagement were not systematically collected as study data elements. Additionally, we did not collect detailed information on site-level utilization of strategies for optimizing diverse recruitment. We therefore were unable to objectively determine and compare the extent to which individual teams utilized different recruitment approaches, thus limiting our insights to estimating the impact of recruitment practices as a bundle. Throughout the 24-week study period, study coordinators and the app team diligently communicated with participants, but the amount of time expended, methods used, and absolute number of outreach interactions initiated by study coordinators to participants was not collected in part due to overburdening study coordinators with administrative work. Future studies could consider collecting these specific data to help clarify which recruitment and retention/engagement tasks are more effective overall, at which study stages, and with which types of participants, though consideration of overall administrative burden placed on the coordination staff must be weighed. Additionally, although retrospective recruitment methods used RUCA 4-Codes derived from home address ZIP Codes to determine rurality, participant rurality at the time of enrollment was self-reported. Participant ZIP Codes were not collected for analysis, thus leaving the potential for a discrepancy between study participants’ perception of their rurality and RUCA categorizations of rurality. Nonetheless, there is no standard definition of rurality across the federal government, and discrepancies exist among various agencies. Self-identified rurality could more accurately reflect the psychosocial factors that contribute to vaccination decisions than RUCA codes [43–47]. We were unable to perform formal inference testing due to differences in population sampling and weighting in the Household PS as compared to the study population enrolled in MoVeUP. Finally, we were unable to perform rigorous inferential statistical analysis of population representativeness due to the lack of uniformity of sampling and weighting strategies between the MoVeUP cohort and the Household Pulse Survey.

## Conclusion

The MoVeUP clinical trial demonstrates that a multi-faceted approach to participant recruitment that employs intentionality in recruiting diverse participants, particularly participants most reflective of the populations of interest, can be effective when employed in primary care settings, including rural communities. Our results can be used to inform future clinical trials design and recruitment/engagement plans for studies conducted in primary care settings.
